# Oridonin Inhibits SARS‐CoV‐2 by Targeting Its 3C‐Like Protease

**DOI:** 10.1002/smsc.202100124

**Published:** 2022-03-13

**Authors:** Baisen Zhong, Weiyu Peng, Shan Du, Bingyi Chen, Yajuan Feng, Xinfeng Hu, Qi Lai, Shujie Liu, Zhong-Wei Zhou, Pengfei Fang, Yan Wu, Feng Gao, Huihao Zhou, Litao Sun

**Affiliations:** ^1^ School of Public Health (Shenzhen) Shenzhen Campus of Sun Yat-sen University Shenzhen 518107 China; ^2^ Laboratory of Pathogen Microbiology and Immunology Institute of Microbiology Chinese Academy of Sciences (CAS) Beijing 100101 China; ^3^ School of Pharmaceutical Sciences Sun Yat-sen University Guangzhou 510006 China; ^4^ School of Medicine Shenzhen Campus of Sun Yat-sen University Shenzhen 518107 China; ^5^ State Key Laboratory of Bioorganic and Natural Products Chemistry Center for Excellence in Molecular Synthesis Shanghai Institute of Organic Chemistry Shanghai 200032 China; ^6^ Department of Pathogen Microbiology School of Basic Medical Sciences Capital Medical University Beijing 100069 China; ^7^ Laboratory of Protein Engineering and Vaccines Tianjin Institute of Industrial Biotechnology Chinese Academy of Sciences (CAS) Tianjin 300308 China

**Keywords:** 3C-like protease, Oridonin, severe acute respiratory syndrome coronavirus 2 (SARS-CoV-2), traditional Chinese medicine, virus inhibitor

## Abstract

The current COVID‐19 pandemic, caused by severe acute respiratory syndrome coronavirus 2 (SARS‐CoV‐2), is an enormous threat to public health. The SARS‐CoV‐2 3C‐like protease (3CLpro), which is critical for viral replication and transcription, has been recognized as an ideal drug target. Herein, it is identified that three herbal compounds, Salvianolic acid A (SAA), (–)‐Epigallocatechin gallate (EGCG), and Oridonin, directly inhibit the activity of SARS‐CoV‐2 3CLpro. Further, blocking SARS‐CoV‐2 infectivity by Oridonin is confirmed in cell‐based experiments. By solving the crystal structure of 3CLpro in complex with Oridonin and comparing it to that of other ligands with 3CLpro, it is identified that Oridonin binds at the 3CLpro catalytic site by forming a C—S covalent bond, which is confirmed by mass spectrometry and kinetic study, blocking substrate binding through a nonpeptidomimetic covalent binding mode. Thus, Oridonin is a novel candidate to develop a new antiviral treatment for COVID‐19.

## Graphical abstract

Oridonin, a natural product extracted from *Rabdosia rubescens*, has been used as a traditional Chinese medicine. Combining biochemistry, cell biology, and crystallography experiments, it is found that Oridonin targets the SARS‐CoV‐2 3CL protease by covalently binding to cysteine145 in its active pocket to exert an anti‐SARS‐CoV‐2 effect.

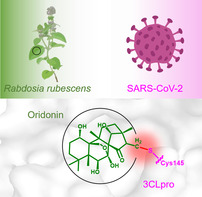

## 1 Introduction

The outbreak of severe acute respiratory syndrome coronavirus 2 (SARS‐CoV‐2) has resulted in a global pandemic of coronavirus disease 2019 (COVID‐19).^[^ ^1^ ^]^ To make matters worse, rapid mutations in the SARS‐CoV‐2 genome and the development of new virulent strains have increased infection and mortality among COVID‐19 patients.^[^ ^2^, ^3^ ^]^ Currently, there are few therapeutic options available for COVID‐19 control. Though various companies and research groups have developed/are developing different types of vaccines for COVID‐19, and drug development is also in progress, only nirmatrelvir has gained an Emergency Use Authorization (EUA) from the Food and Drug Administration (FDA) for the treatment of patients with mild‐to‐moderate COVID‐19 thus far.^[^ ^4^ ^]^

SARS‐CoV‐2 is an enveloped, positive‐sense single‐stranded RNA virus containing a 30 kb genome that encodes two cysteine proteases, papain‐like protease (PLpro) and 3C‐like protease (3CLpro), that mediate viral replication and transcription.^[^ ^5^ ^]^ The 3CLpro is responsible for cleaving polyproteins at 11 cleavage sites yielding NSP4‐9 and NSP12‐15. The released NSPs form the viral RNA polymerase complexes, which are involved in replication and transcription of newly formed virus in the host.^[^ ^6^ ^]^ Due to its vital function in the SARS‐CoV‐2 life cycle and the absence of homologous proteins in humans, 3CLpro is recognized as an ideal drug target for high‐throughput screening of compounds or fragments to devise effective inhibitors for SARS‐CoV‐2.^[^ ^7^, ^8^ ^]^ Indeed, a series of SARS‐CoV‐2 3CLpro inhibitors have been reported, but most of them are substrate‐like peptidomimetic inhibitors with similar binding mode in the catalytic pocket, none of which are suitable for clinical application yet.^[^ ^9^, ^10^ ^]^

Plant‐based small molecules have been developed for thousands of years and display efficacy against different diseases and ailments, including infectious disease.^[^ ^11^ ^]^ In current investigations to identify safe and efficacious drugs for COVID‐19, natural products with a range of valuable bioactivities have attracted widespread attention in terms of evaluating and validating their therapeutic effects, especially as starting points in the development of therapeutic strategies. Here, we identified three compounds, Salvianolic acid A (SAA), (–)‐Epigallocatechin gallate (EGCG), and Oridonin, that inhibit the activity of SARS‐CoV‐2 3CLpro. Further, we demonstrated that Oridonin blocks the infectivity of SARS‐CoV‐2 in Vero E6 cell‐based experiments. Solving the cocrystal structure of the 3CLpro‐Oridonin complex revealed that Oridonin binds at the 3CLpro catalytic site by forming a C—S covalent bond, and blocks substrate binding. In summary, we report that Oridonin has good inhibitory activity against SARS‐CoV‐2 at the molecular and cellular levels using biochemical, biophysical, and crystallographic methods. This work both identified a novel herbal compound Oridonin to treat SARS‐CoV‐2 and established its inhibitory mechanism, providing critical information for the design and optimization of nonpeptidomimetic covalent inhibitors of SARS‐CoV‐2 3CLpro.

## 2 Results

### 2.1 Screening for Potential 3CLpro Inhibitors

**Figure 1:**
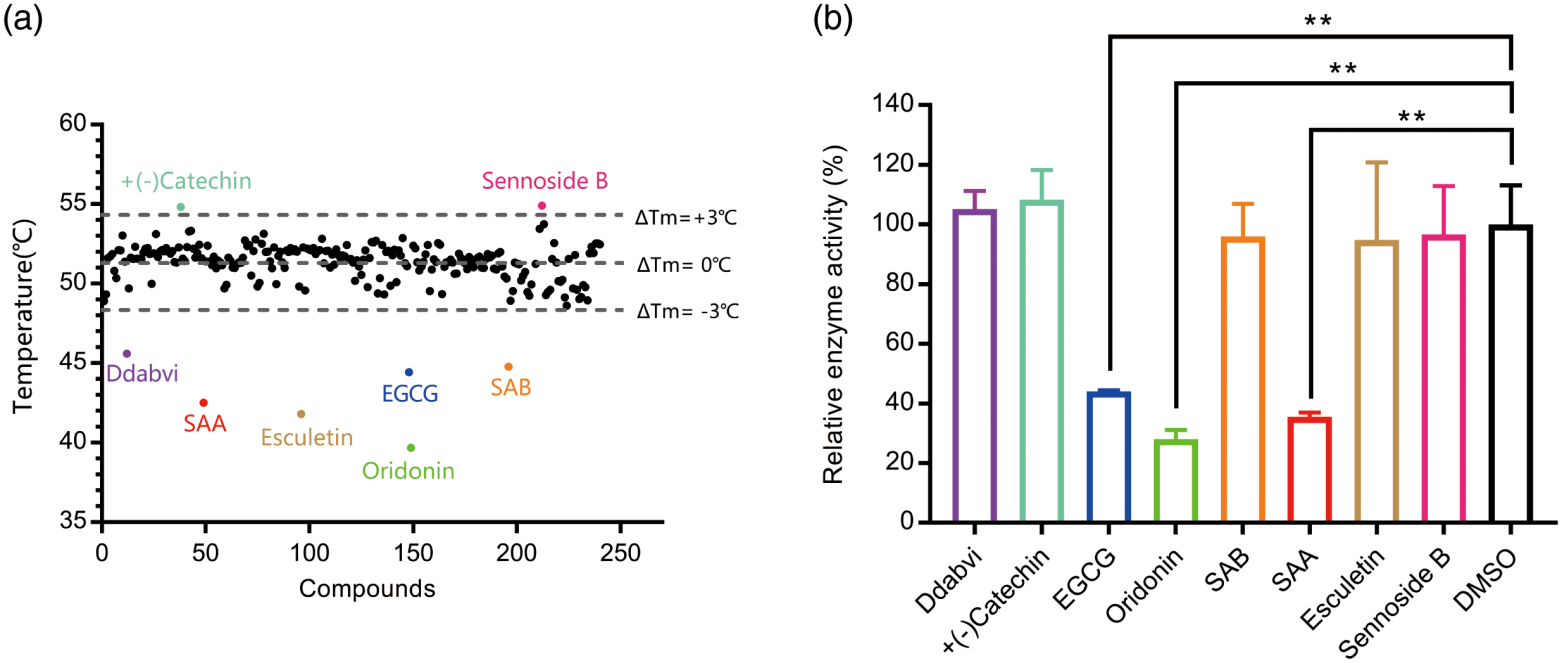
Screening for 3CLpro inhibitors based on the thermal shift assay. a) Eight compounds shift the melting temperature of 3CLpro by >3.0 °C and are considered as positive hits among 237 tested compounds. Black points within gray dashed lines represent negative hits. b) Verification of the inhibitory effect of eight positive hits from TSA versus the DMSO control (*n* = 2 independent replicates, *p* = 0.0081, 0.0015, and 0.0032 for EGCG, Oridonin, and SAA, respectively; not significant *p* > 0.05, * *p* < 0.05, and ** *p* < 0.01).

Given our goal of identifying potential usable and practical drugs against SARS‐CoV‐2, we purified recombinant SARS‐CoV‐2 3CLpro and used it as the bait to perform fluorescence‐based thermal shift assay (TSA). In this assay, as the temperature increases, the protein undergoes thermal unfolding and exposes its hydrophobic core, at which point a fluorescent dye binds to the hydrophobic regions and becomes unquenched. Fluorescence is monitored, and the midpoint of the protein unfolding transition is defined as the *T*_m_. Ligand binding usually influences protein thermal stability during the denaturation process, and the ligand‐binding affinity of any potential inhibitor can be assessed from the shift of the *T*_m_.^[^ ^12^ ^]^ Based on this, 237 tested compounds consisting of traditional Chinese herbal medicine, natural bioactive products, and marine active compounds from our homemade library were screened with this assay. The *T*_m_ of 3CLpro in the absence of any compounds was 51.2 ± 0.2 °C (the standard deviation was calculated from three repeats), and was used as control. Compounds were considered as positive hits when they changed the *T*_m_ value of 3CLpro by >3.0 °C. Ultimately, eight positive hits were identified (Figure 1a) and subsequently subjected to enzymatic assays.

### 2.2 Inhibition of the Enzymatic Activity of 3CLpro by the Selected Compounds

To further confirm whether the eight positive hits have an inhibitory effect on SARS‐CoV‐2 3CLpro, a fluorescence resonance energy transfer (FRET) protease assay was applied.^[^ ^13^ ^]^ We used purified 3CLpro to hydrolyze a peptide substrate, which was fluorescently labeled with a FRET pair and can be monitored when it is cut by 3CLpro. The verification experiments were performed with 1.2 μm 3CLpro and 50 μm of the positive hit compounds to determine the inhibitory effects of the compounds on 3CLpro. EGCG, SAA, and Oridonin displayed significant inhibition of 3CLpro activity, while the other five had no effect (Figure 1b) (**Table** **1**).

**Table 1:** The eight positive hits of thermal shift assay and their inhibitory effect on 3CLpro

Compound	Structure	Δ*T*_m_ [°C]a)	Relative enzyme activity [%]b)
+(–)Catechin	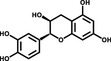	3.2	105
Sennoside B	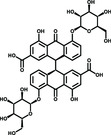	3.0	97
Ddabvi	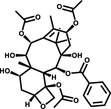	−5.1	108
EGCG	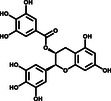	−6.3	44
SAB	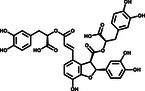	−6.4	96
SAA	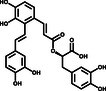	−8.3	36
Esculetin	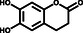	−9.4	95
Oridonin	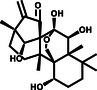	−11.1	28

a) Δ*T*_m_ is the shift between the Δ*T*_m_ values of 3CLpro with and without compounds.

b) Relative enzyme activity represents the ratio of the enzymatic activity of 1.2 μmol 3CLpro at 50 μmol compound. The data are the average of two independent experiments.

**Figure 2:**
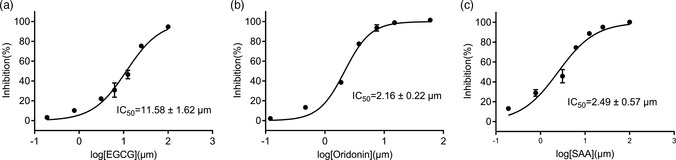
Three compounds inhibit the catalytic activity of 3CLpro in vitro. Assays containing a) EGCG, b) Oridonin, and c) SAA show efficient inhibitory activity of 3CLpro in vitro. Error bars represent the mean ± SD of three independent experiments.

To further evaluate the inhibitory activities of the three inhibitors, we examined their impact on enzyme kinetics. EGCG inhibited 3CLpro with an IC_50_ of 11.58 μm, while SAA and Oridonin demonstrated inhibitory activities with IC_50_ values of 2.49 and 2.16 μm, respectively (**Figure** **2**a–c). The inhibitory activities of these three compounds are consistent with the TSA results, as Oridonin caused a larger *T*_m_ shift than the other two compounds. EGCG, the most abundant component of catechins in tea (*Camellia sinensis* (L.) O. Kuntze), has anticancer, antioxidant, anti‐inflammatory, anti‐collagenase, and antifibrosis effects.^[^ ^14^ ^]^ SAA is extracted from *Salvia miltiorrhiza* (Danshen), an important traditional Chinese medicinal herb, that has been used in China for the treatment of cardiovascular diseases for hundreds of years.^[^ ^15^ ^]^ Oridonin, the main bioactive chemical component of *Rabdosia rubescens* (Hemsl.) H.Hara, a perennial herb widely used in China against inflammation, bacterial infections, respiratory and gastrointestinal diseases, and cancers, is a tetracyclic diterpenoid compound.^[^ ^16^ ^]^ Taken together, both the TSA and enzyme inhibition assay revealed that SAA, Oridonin, and EGCG can directly bind to and effectively inhibit SARS‐CoV‐2 3CLpro. Interestingly, the compounds SAA and EGCG are reported to block viral infection by binding to both the viral receptor‐binding domain (RBD) and the host cell ACE2 receptor,^[^ ^17^, ^18^ ^]^ indicating that both compounds could inhibit SARS‐CoV‐2 through multiple mechanisms.

### 2.3 Oridonin Directly Binds to 3CLpro via Covalent Bond Formation

**Figure 3:**
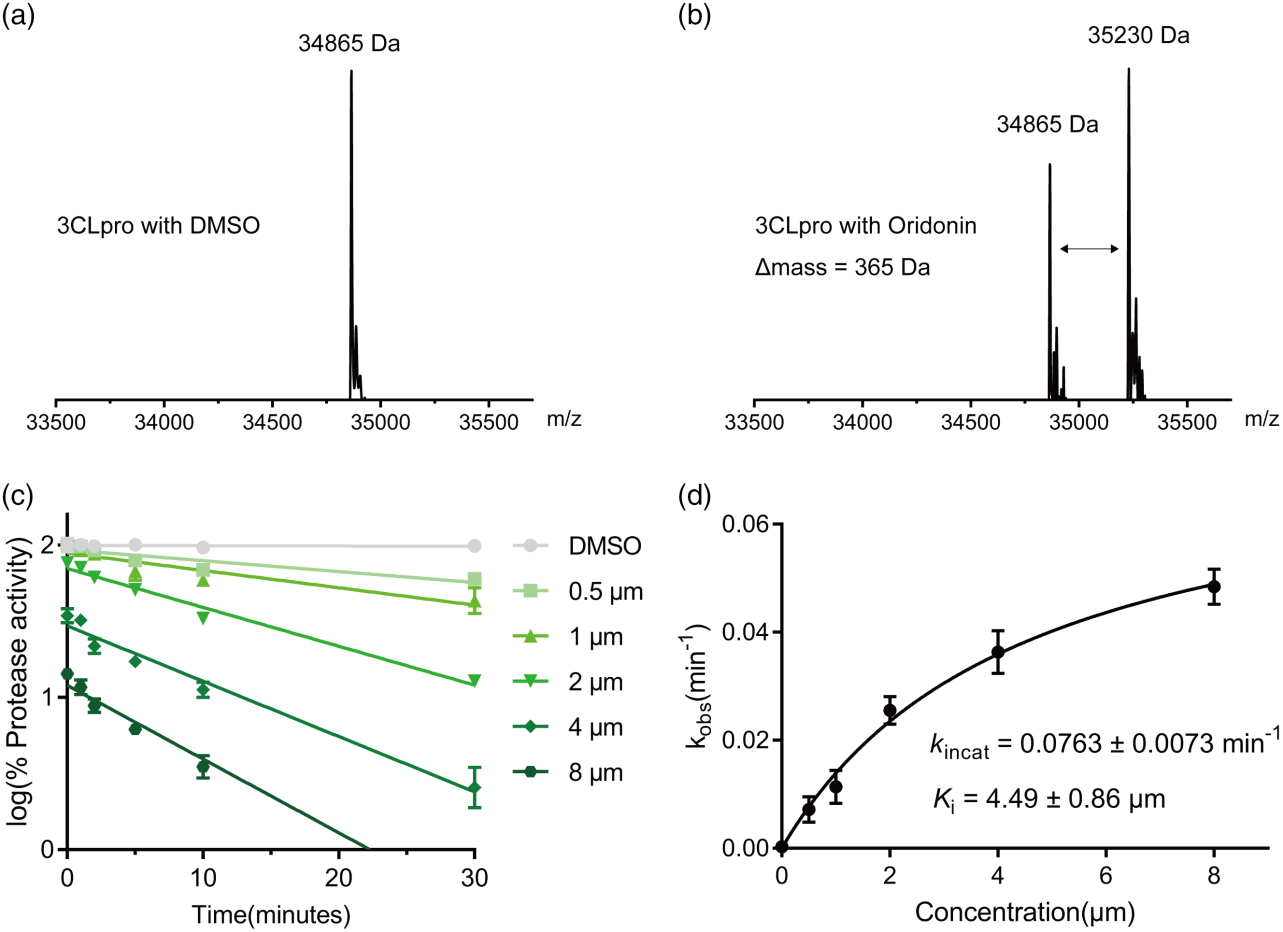
Mass spectrometry analysis and kinetic studies of 3CLpro by Oridonin. a) The molecular ion profile of 3CLpro treated with DMSO. b) The molecular ion profile of 3CLpro treated with Oridonin. The theoretical molecular mass is 34 865 Da (average mass) for 3CLpro and 35 230 Da (average mass) for 3CLpro treated with Oridonin. The Δ mass is equal to the molecular mass of Oridonin. c) The 3CLpro was incubated with five different concentrations of Oridonin, respectively. For the protease activities of each concentration at different incubation time was measured by the FRET protease assay and plotted against different incubation time. d) The resulting *k*_obs_ values were plotted versus Oridonin concentrations to calculate the *k*_inact_ and *K*_i_ values.

Oridonin displayed the best inhibitory activity and the largest thermal shift, compared to the other two compounds. *T*_m_ shifts to lower temperatures are reportedly associated with an apparent destabilization of the protein by covalently bound compounds, such as in the case of the SARS‐CoV‐2 3CLpro inhibitor GRL‐1720.^[^ ^19^ ^]^ Also, Oridonin has an *α*,*β*‐unsaturated olefin group, which has been reported to covalently bind to a cysteine residue.^[^ ^20^ ^]^ To determine if Oridonin forms a covalent bond with 3CLpro, 3CLpro was incubated with or without Oridonin for 2 h. Then, an electrospray ionization mass spectrometry (ESI‐MS) experiment was performed to detect the mass of 3CLpro. Compared to the mass of 3CLpro alone, the mass in the presence of Oridonin increased by 365 Da, which is consistent with the molecular weight of a molecule of Oridonin (**Figure** **3**a,b). We further characterized Oridonin by conducting enzyme kinetic studies to determine the inactivation rate constant (*k*_inact_) and the equilibrium‐binding constant (*K*_i_). Time‐dependent inhibition by Oridonin was observed (Figure 3c), and the relationship between Oridonin concentrations and *k*_obs_ resulted in the *k*_incat_ of 0.0763 min^−1^ and the *K*_i_ of 4.49 μm (Figure 3d). These data suggest that Oridonin binds to 3CLpro through a covalent bond.

### 2.4 Oridonin Inhibits the Replication of SARS‐CoV‐2 in Vero E6 Cells

**Figure 4:**
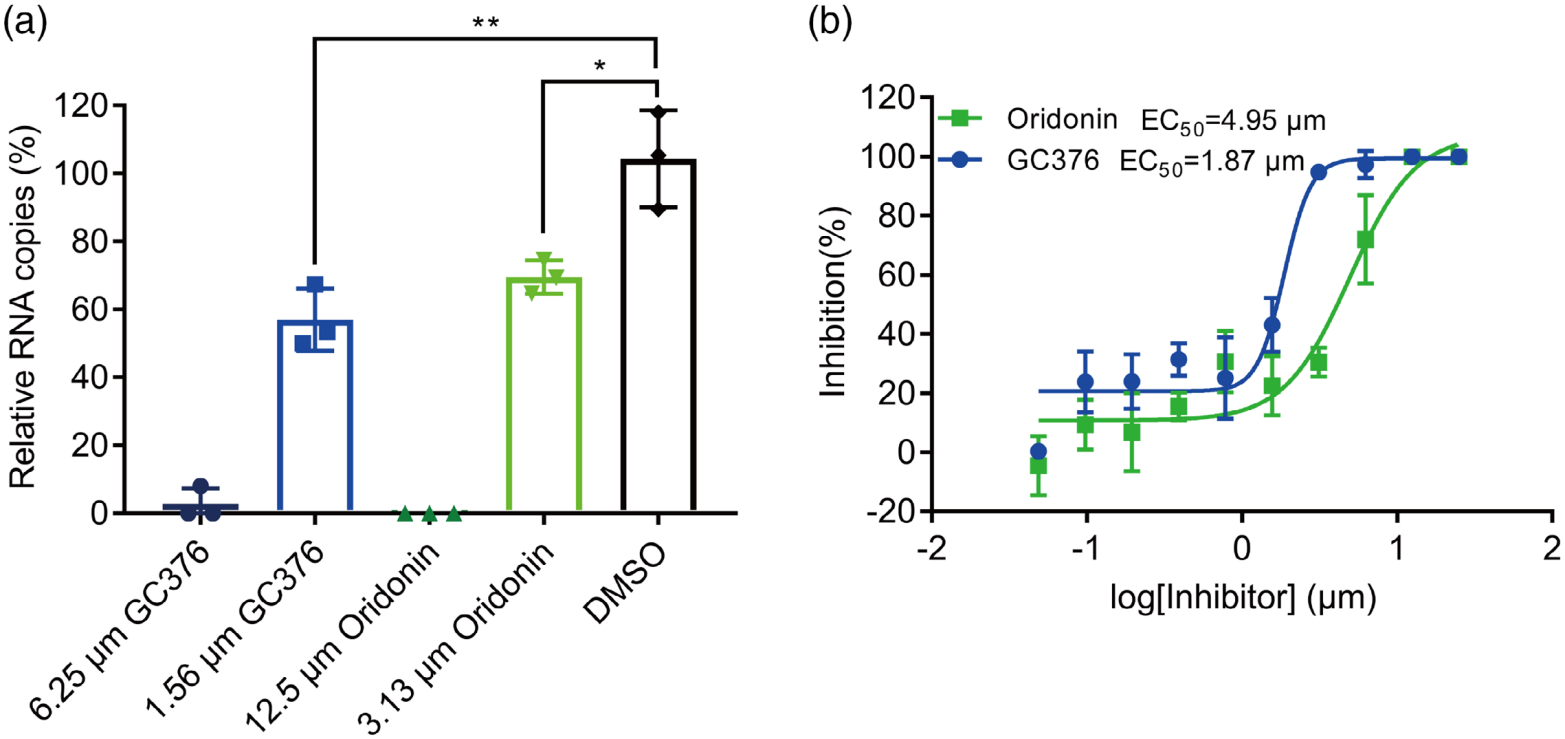
The antiviral activity of Oridonin against SARS‐CoV‐2. a) The relative copies of viral RNA present in the supernatant at 48 h postinfection determined by real‐time quantitative PCR analysis after Oridonin treatment; GC376 was used as a positive control. Unpaired *t*‐tests were used to compare the relative viral RNA copies incubated with inhibitor versus the DMSO control (*n* = 3 independent replicates; *p* = 0.0031 and 0.0137 for 1.56 μm GC376 and 3.13 μm Oridonin, respectively; not significant *p* > 0.05, * *p* < 0.05, and ** *p* < 0.01). b) The inhibitory effect of Oridonin and GC376 treatment on SARS‐CoV‐2 replication. GC376 was used as a positive control.

We further evaluated the antiviral efficacy of Oridonin against SARS‐CoV‐2 in Vero E6 cells. We infected the cells with SARS‐CoV‐2 at a multiplicity of infection (MOI) of 0.01 in the presence of a series of gradient concentrations of Oridonin. Then, quantitative real‐time RT‐PCR (qRT‐PCR) was used to evaluate antiviral efficacy by quantification of viral RNA copy numbers in the cell supernatants. We found that Oridonin inhibits SARS‐CoV‐2 in a dose‐dependent manner **(** **Figure** **4**a **)**, with a half‐maximal effective concentration (EC_50_) of Oridonin against SARS‐CoV‐2 at 4.95 μm. As a positive control, GC376, an effective 3CLpro inhibitor, also decreased the SARS‐CoV‐2 RNA copy number in Vero E6 cells compared to the DMSO control, with an EC_50_ of 1.87 μm **(**Figure 4b **)**.^[^ ^21^ ^]^ Subsequently, the cytotoxicity of Oridonin in Vero E6 cells was measured via the CCK‐8 assay, and the half‐cytotoxic concentration (CC_50_) was 24.94 μm (Figure S1, Supporting Information). The resulting selectivity index (SI = CC_50_/CE_50_) value is >5 for Oridonin. Taken together, these cell‐based antiviral results indicate that Oridonin prevents the replication of SARS‐CoV‐2 via inhibition of 3CLpro.

### 2.5 Crystal Structure of the 3CLpro‐Oridonin Complex

**Figure 5:**
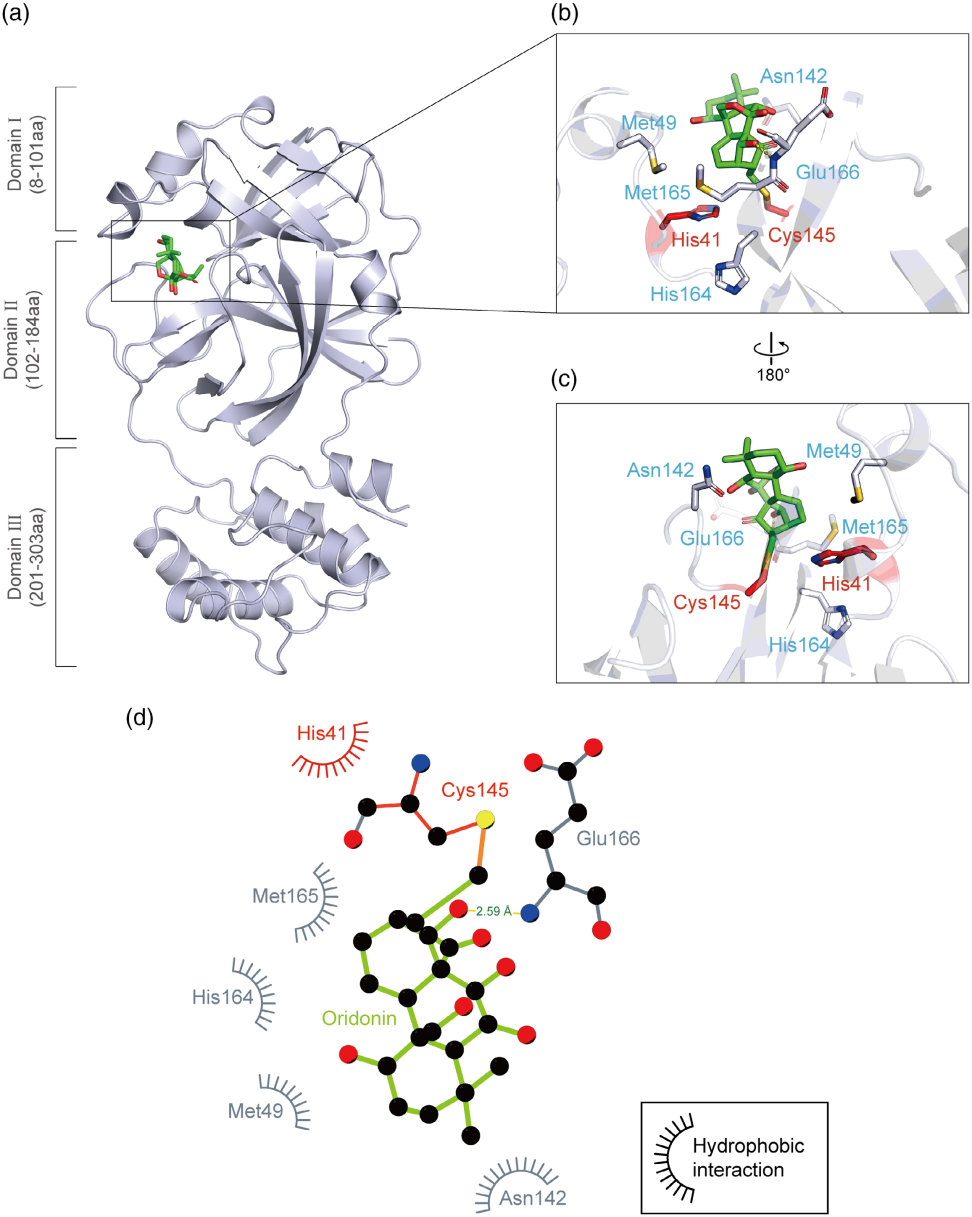
Crystal structure of SARS‐CoV‐2 3CLpro in complex with Oridonin. a) Overview of the structure of Oridonin‐bound 3CLpro. The protein is shown as a cartoon in blue‐white and Oridonin is presented as green sticks. b–c) Zoomed‐in view of the binding site of Oridonin. The catalytic residues His41 and Cys145 are shown in red sticks, and others residues interacting with Oridonin are shown in blue‐white sticks. The hydrogen bonds formed with the residues of 3CLpro are indicated as yellow dashed lines. d) 2D presentation of Oridonin binding. The ligand Oridonin and the residues forming polar interactions are shown in ball‐and‐stick representations, the C—S covalent bond is shown in orange, the hydrogen bond is shown as a yellow line with the distance labeled, and other residues contribute to hydrophobic interactions.

To elucidate the inhibitory mechanisms of Oridonin, we determined the crystal structure of 3CLpro in complex with Oridonin at a resolution of 2.10 Å. In agreement with previous studies, 3CLpro formed a homodimer, and each molecule is composed of three domains (domains I, II, and III) and an extended loop region, connecting domains II and III. The substrate‐binding pocket lies in the cleft between domains I and II (**Figure** **5**a).^[^ ^22^ ^]^ Clear electron densities of Oridonin were observed in the classic substrate‐binding pockets of both 3CLpro molecules. The unambiguous omit map clearly shows that the carbon atom of the Oridonin olefin group forms a C—S covalent bond with the Sγ atom of Cys145 of 3CLpro, corroborating that Oridonin forms a covalent bond with 3CLpro (Figure S2, Supporting Information). In the active site of 3CLpro, the sulfur atom from Cys145 gains nucleophilicity, and presumably exerts a nucleophilic attack on the electrophilic carbon atom of the *α*,*β*‐unsaturated olefin group of Oridonin. In addition to the C—S covalent bond, the inhibitor is also stabilized by a hydrogen bond between the hydroxyl group of Oridonin and the backbone nitrogen of Glu166 in 3CLpro (Figure 5b,c). Hydrophobic interactions are mainly contributed by the side chains of His41, Met49, Asn142, His164, and Met165 (Figure 5d).

**Figure 6:**
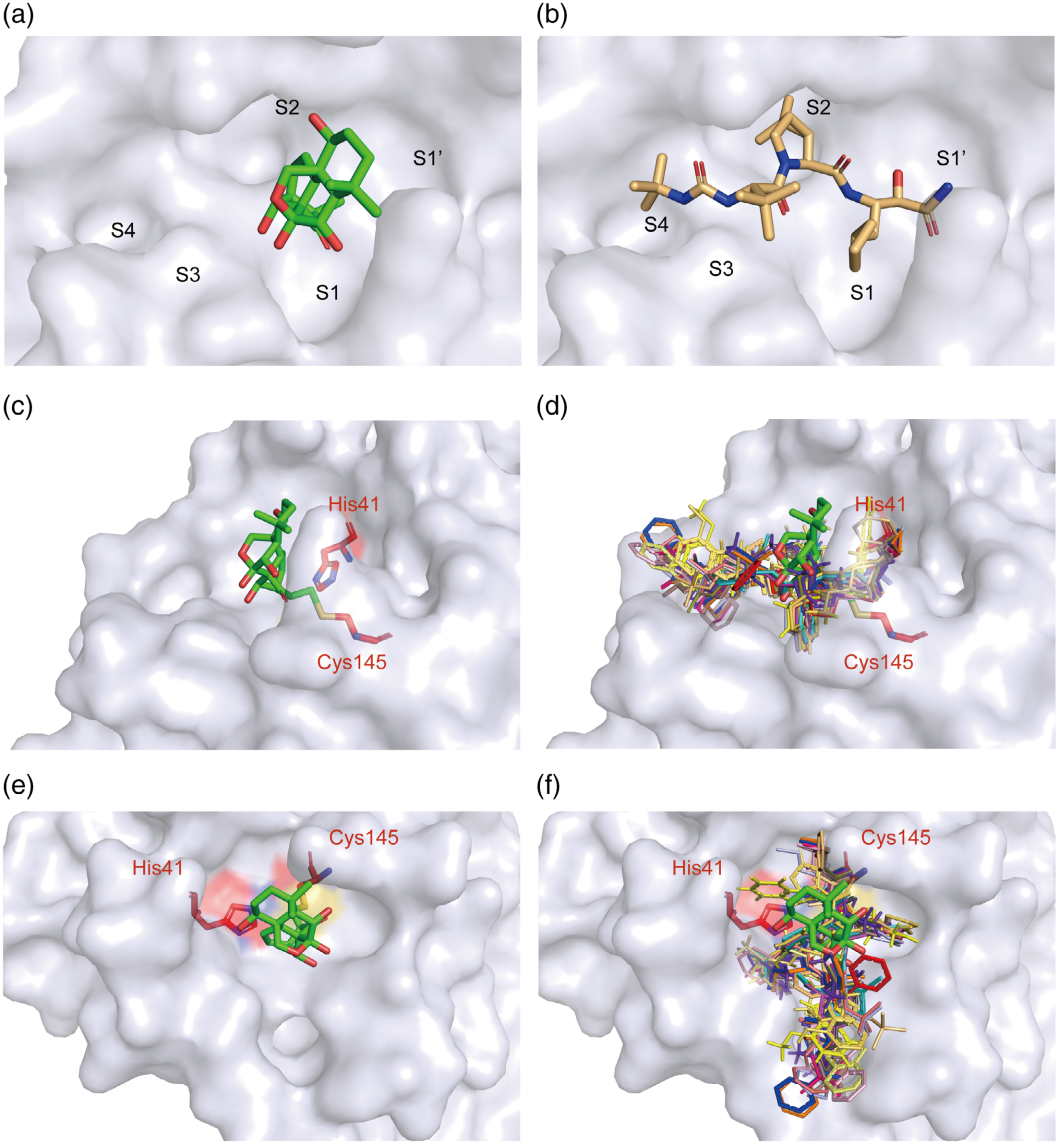
Binding mode of Oridonin with 3CLpro. a) Oridonin only occupies the S1 of the substrate‐binding pocket. b) Boceprevir occupies the S1, S2, S3, and S4 subsites of the substrate‐binding pocket. c) The structure of 3CLpro is shown in surface representation in blue‐white, Oridonin is shown as green stick, and the two catalytic residues are shown as red sticks. d) The known structures of 3CLpro bound to inhibitors are superimposed on the 3CLpro‐Oridonin complex. Different colors represent reported inhibitors, which were shown as lines (PDB codes: 7D1M, 7D10, 7C8T, 7C7P, 7C8R, 7COM, 7JKV, 6W63, 5RGY, 5RH5, 6Y2F, 6XHM, 7BRP, 5RH6, and 5RH7). e,f) The top views of (c) and (d), respectively.

A “catalytic water” coordinated by His41, His164, and Asp187 plays a key role in the catalysis of 3CLpro.^[^ ^19^ ^]^ Similar to most 3CLpro‐inhibitor complex structures, this water is observed in the 3CLpro‐Oridonin complex, and Oridonin does not have to compete with this conserved “catalytic water” to inhibit SARS‐CoV‐2 3CLpro (Figure S3a,b, Supporting Information). However, most inhibitors usually have to simultaneously occupy multiple subsites to achieve high binding affinity (e.g., Boceprevir occupies the S1, S2, S3, and S4 subsites of the active pocket), while Oridonin only binds to the S1 subsite (Figure 6a,b, Supporting Information).^[^ ^21^ ^]^

### 2.6 Inhibitory Mechanism of Oridonin Against 3CLpro

We collected all of the available crystal structures of SARS‐CoV‐2 3CLpro bound to different ligands from the Protein Data Bank (PDB), and found that there are three main binding positions presented in 3CLpro, including position A, the catalytic site (shown bound to Boceprevir); position B, the cleft between domains I and II (the opposite side of position A, shown bound to Tegafur); and position C, a surface groove of domain III (shown bound to Ifenprodil) (Figure S4a–c, Supporting Information). We then focused on the chemical structures of ligands bound in all three binding positions. It is clear that the ligands lying in position A employ a common chemical structural scaffold compared to the ligands bound in the other two positions (Table S1, Supporting Information). Interestingly, the amino acid sequences of the substrates recognized by 3CLpro are highly conserved and the common chemical scaffold exactly matches the structure of the substrate proteolysis site (Figure S5a,b, Supporting Information), indicating that they serve as peptidomimetic inhibitors of 3CLpro.^[^ ^23^ ^]^ In contrast, Oridonin is positioned slightly outward compared to other compounds, and functions as a “barrier” in front of the active sites to effectively prevent the substrate from entering the catalytic pocket (Figure 6c–f).

**Figure 7:**
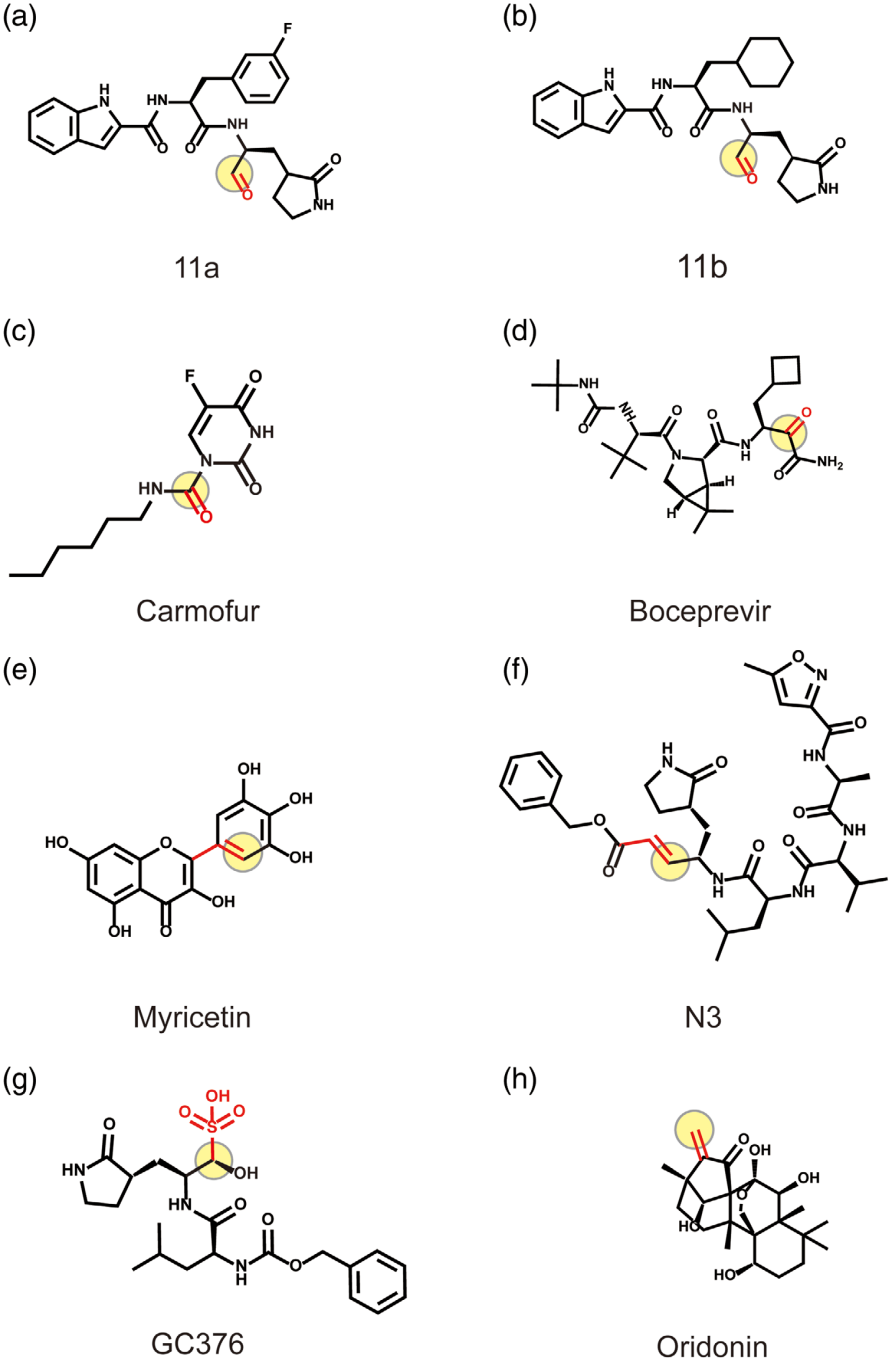
Covalent binding inhibitors of 3CLpro and their electrophilic group. Oridonin and other reported covalent binding inhibitors. The red functional groups represent the electrophilic groups that covalently bind to Cys145, and the light‐yellow circles represent the atomic sites that form the covalent bond. a,b) The aldehyde group of 11a and 11b. c,d) The carbonyl group of Boceprevir and Carmofur. e) The pyrogallol group of Myricetin. f) The acyclic peptide structure *α*,*β*‐unsaturated ester group of N3. g) The aldehyde precursor group of GC376. h) The *α*,*β*‐unsaturated olefin group of Oridonin.

Cys145 has a nucleophilic sulfhydryl group in the active site of 3CLpro, which is the target of many covalent binding inhibitors. The reported covalent binding inhibitors and Oridonin all covalently bind to the sulfhydryl group of Cys145 (Figure S6a–h, Supporting Information). Among them, 11a and 11b are reported to form covalent bonds between the electrophilic aldehyde group and the thiol group of 3CLpro Cys145, different from the carbonyl group of Boceprevir and Carmofur (**Figure** **7**a–d).^[^ ^21^, ^24^, ^25^ ^]^ Myricetin forms a covalent bond with Cys145 through the *α*,*β*‐unsaturated carbonyl group electrophilic acceptor of *o*‐quinone from oxidized pyrogallol (Figure 7e).^[^ ^26^ ^]^ N3 also uses an acyclic peptide structure *α*,*β*‐unsaturated ester group to covalently binds to Cys145 (Figure 7f).^[^ ^22^ ^]^ GC376 creates a covalent bond by removing the sulfonic acid group and forms an aldehyde group.^[^ ^21^ ^]^ Different from the above compounds, Oridonin, as a kaureane skeleton compound, has the activity of an *α*,*β*‐unsaturated olefin group, is a classic enone Michael electrophilic acceptor, and covalently binds to the thiol group of Cys145 (Figure 7h). The C—S covalent bond formed specifically with Cys145 is an important mechanism for Oridonin to inhibit 3CLpro.

Taken together, the nonpeptidomimetic covalent binding mode of Oridonin with SARS‐CoV‐2 3CLpro indicates a different inhibition mechanism than previously reported for other inhibitors. Thus, Oridonin is a novel candidate for further development of SARS‐CoV‐2 3CLpro covalent inhibitors.

## 3 Discussion

COVID‐19 is an ongoing global health crisis and has caused a serious health threat worldwide. With the rapid mutation of SARS‐CoV‐2, the emergence of many variants has gradually made previous vaccines and antibodies ineffective.^[^ ^3^ ^]^ To date, the FDA has only approved an EUA for ritonavir‐boosted nirmatrelvir (Paxlovid) for the treatment of patients with mild‐to‐moderate COVID‐19.^[^ ^4^ ^]^ SARS‐CoV‐2 3CLpro is a promising therapeutic target because it plays an important role in the life cycle of the virus and is relatively conserved among different variants.^[^ ^6^ ^]^

Here, we first demonstrate that Oridonin, the major active constitute of *Isodon rubescens* (Hemsl.) H.Hara, a commonly used traditional Chinese medicine for the treatment of inflammatory diseases,^[^ ^16^ ^]^ directly and covalently binds to SARS‐CoV‐2 3CLpro and has remarkable antiviral activity both in vitro and in vivo. This suggests that Oridonin can be used as a lead to design new therapeutics against COVID‐19 diseases.

During our initial screen, we performed the TSA assay as the first step to identify potential 3CLpro inhibitors. In addition to SAA, EGCG, and Oridonin, we found several other candidates that also caused a shift of the thermal stability of SARS‐CoV‐2 3CLpro. However, when we tested their effects on enzymatic activity, the compounds showed very low or no inhibition activities, suggesting that the compounds had low affinity or may not bind in the catalytic pocket. Two of the three identified inhibitors, SAA and EGCG, are reported to be effective in inhibiting SARS‐CoV‐2‐induced cytopathic effect and plaque formation in Vero E6 cells. Both SAA and EGCG can bind to the viral RBD and host cell ACE2 receptor, which blocks viral infection.^[^ ^17^, ^18^ ^]^ EGCG is also known to inhibit 3CLpro activity.^[^ ^27^ ^]^ Our results indicate that SAA also directly targets 3CLpro and blocks its enzymatic activity, suggesting that these two compounds may inhibit SARS‐CoV‐2 infection in several ways by targeting the RBD, ACE2, and/or 3CLpro.

Interestingly, in the cocrystal structure, Oridonin creates a C—S covalent bond with Cys145 in 3CLpro, which were verified by ESI‐MS and enzyme kinetic studies. Many covalent and noncovalent SARS‐CoV‐2 3CLpro inhibitors have recently been reported, and their binding modes have been determined by high‐resolution structures.^[^ ^8^, ^25^ ^]^ However, some of them have only been captured in crystal structures, with no associated enzymatic activity tests. Further, for those evaluated in vitro, it is not known whether they can inhibit the replication of live virus in cells or in animals.^[^ ^28^, ^29^ ^]^ Also, most of them are essentially peptidomimetic inhibitors, such as Boceprevir and GC376, which inhibit 3CLpro activity by occupying its substrate binding pocket. Oridonin forms a C—S covalent bond with the S*γ* atom of Cys145 and a hydrogen bond with the main chain of Glu166 from 3CLpro. The amino acids around the ligand (His41, Met49, Asn142, His164, and Met165) mainly contribute hydrophobic interactions. Despite safety concerns due to the potential risk of idiosyncratic toxicity or drug hypersensitivity, covalent drugs have been approved as treatments for diverse clinical indications, have made a major positive impact on human health, and they can provide some pharmacological advantages, including enhanced potency and prolonged duration of action.^[^ ^30^, ^31^ ^]^ Based on the inhibition mechanism, the *α*, *β*‐unsaturated olefin group of Oridonin can work as a warhead that can covalently link to Cys145 of 3CLpro for the future development of antiviral drugs for SARS‐CoV‐2.

## 4 Conclusion

In summary, we showed that Oridonin can inhibit the replication of SARS‐CoV‐2 in Vero E6 cells via inhibition of 3CLpro with an EC_50_ of 4.95 μm. Oridonin or its derivatives might be promising candidates to develop anti‐SARS‐CoV‐2 agents. Moreover, combination therapy using a different class of agents (e.g., a mixture of a 3CLpro inhibitor, a PLpro inhibitor, and a RdRp inhibitor) might be a promising therapeutic strategy for the treatment of SARS‐CoV‐2 infection.

## 5 Experimental Section

5.1

5.1.1

##### Expression and Purification of SARS‐CoV‐2 3CLpro

The full‐length cDNA of SARS‐CoV‐2 3CLpro (GenBank: MN908947.3) was synthesized and codon‐optimized before being cloned into the pET20b vector (Novagen) with a 6xHis tag at its C‐terminus. After transformation with the constructed plasmid, BL21 (DE3) *Escherichia coli* cells (Novagen) were grown in Luria–Bertani (LB) medium supplemented with 100 mg mL^−1^ ampicillin at 37 °C until the optical density at 600 nm (OD_600_) reached 0.6–0.8. Then, isopropyl‐β‐d‐thiogalactopyranoside (IPTG) was added at a concentration of 500 μmol to induce protein overexpression, and cells were further cultured at 16 °C for approximately 22 h. The cells were collected by centrifugation at 4200 rpm for 45 min, and the cell pellets were resuspended in lysis buffer (Tris‐HCl pH 8.0 (20 mmol), NaCl (300 mmol), and imidazole (5 mmol)), lysed in a low temperature and high‐pressure cell crusher, and then centrifuged at 25 000 g for 45 min. Subsequently, the supernatant was loaded onto a Ni‐NTA column (GE Healthcare) pre‐equilibrated with lysis buffer. The column was washed with 10 column volumes of washing buffer (NaCl (300 mmol), Tris‐HCl pH 8.0 (20 mmol), and imidazole (40 mmol)), and then eluted with elution buffer (NaCl (300 mmol), Tris‐HCl pH 8.0 (20 mmol), and imidazole (250 mmol)). The protein was further purified over a HiTrap Q HP column (GE Healthcare) and by size‐exclusion chromatography using a Superdex 200 Increase 10/300 GL column (GE Healthcare) equilibrated with running buffer (Tris‐HCl pH 8.0 (20 mmol), and NaCl (150 mmol)). All purification steps were performed at 4 °C or on ice. The 3CLpro protein was concentrated and stored at −80 °C before use. The protein purity was assessed by SDS‐PAGE.

##### Inhibitor Screening by Fluorescence‐Based TSA

The binding of 237 compounds to 3CLpro was evaluated using a fluorescence‐based TSA. The reactions (20 μL) consisted of 3CLpro (0.2 mg mL^−1^), 1% v/v DMSO, 100 μm of the tested compounds, and 4xSYPRO orange fluorescence dye (Sigma‐Aldrich) in buffer (Tris‐HCl pH 8.0 (20 mmol) and NaCl (150 mmol)) and were prepared in 96‐well PCR plates (Nest Technologies) on the ice. The mixture without any compound was used as a blank control. The 96‐well PCR plates were placed in a StepOne Plus Real‐time PCR system (Life Technologies), incubated at 25 °C for 10 min, and gradually heated from 25 °C to 95 °C at a rate of 1 °C min^−1^. The fluorescence signal of SYPRO orange at 490/530 nm excitation/emission wavelengths during protein thermal denaturation was recorded by the instrument every 30 s. The melting curves (fluorescence intensity versus temperature) were fitted by the StepOne software v2.3 to measure the melting temperature (*T*_m_) of the protein. Triplicate experiments were performed. The Δ*T*_m_ is the shift in the value between the melting temperatures of 3CLpro with compound and the blank control.

##### The Inhibition Assay of SARS‐CoV‐2 3CLpro

The fluorogenic substrate Dabcyl‐TSAVLQ↓SGFRKMK‐Edans was synthesized by GL Biochem (Shanghai, China).^[^ ^21^ ^]^ The experiment process was as follows: recombinant SARS‐CoV‐2 3CLpro (1.2 or 0.5 μmol final concentration) was mixed with serial dilutions of each compound in 80 μL assay buffer (Tris‐HCl pH 8.0 (20 mmol) and NaCl (150 mmol)) on ice, and the reaction system with DMSO was used as a blank control. The reactions were initiated by adding the above fluorogenic substrate (20 μL) to a final concentration of 50 μmol. Then, the fluorescence signal value at 340 nm (excitation)/490 nm (emission) was immediately measured every 30 s for 25 min at 37 °C with a Multi‐Mode Detection Platform (Synergy H1, TioTek, USA). The first 15 min of change in the fluorescence value was used to calculate the reaction velocity, and the initial reaction velocity of different compound concentrations was divided by the reaction velocity of the blank control to calculate the inhibition rate or relative enzyme activity. For inhibitory compounds, three independent experiments were performed for the determination of IC_50_ values. The IC_50_ values are expressed as the mean ± SD and determined via nonlinear regression analysis using GraphPad Prism software 7.0 (GraphPad Software, San Diego, CA, USA). Enzyme kinetic study was designed as previously described.^[^ ^32^ ^]^ Oridonin at various concentrations was preincubated with 3CLpro at different timepoints to further generate *k*_obs_ which were then used to calculate *k*_inact_ and *K*_i_ by GraphPad Prism.

##### ESI‐MS Analysis

The binding of 3CLpro and Oridonin was analyzed by ESI‐MS. First, 3CLpro (10 μmol) and Oridonin (100 μmol) in Tris‐HCl buffer pH 8.0 (20 mmol) were incubated on ice for 2 h. The samples consisting of 3CLpro (10 μmol) and Oridonin (100 μmol) or DMSO were buffer‐exchanged with ammonium acetate (AA) (10 mmol) using Millipore centrifugal filters with a 10 kDa cutoff. The desalted protein samples were further diluted with 49.5/49.5/1 v/v/v methanol/water/formic acid solution for denatured ESI‐MS analysis. 3CLpro aliquots (3 μL) were analyzed using a quadrupole ion mobility time‐of‐flight mass spectrometer (Synapt G2‐Si HDMS) in the positive ion mode, and 3CLpro ions were generated by nano‐ESI from a homemade borosilicate capillary emitter (1.0 mm o.d./0.58 mm i.d.) with a tip i.d. of ≈1 μm pulled using a P‐97 pulle. The detailed instrumental parameters were as follows: capillary voltage 0.7 kV, extraction cone 80 V, sampling cone 100 V, and source temperature 30 °C.

##### Protein Crystallization and Structure Determination

The purified recombinant protein was concentrated to 9 mg mL^−1^ and crystalized by the sitting‐drop vapor‐diffusion method. Crystals of 3CLpro alone were grown in 0.1 mol MES pH 6.0, and 10% PEG 6000 at 16 °C for 3–5 days. The crystals were soaked in the crystallization solution supplemented with compounds (20 mmol) and incubated for 10 min. After incubation, crystals were immersed in a cryoprotectant solution (0.1 mol MES pH 6.0, 10% PEG 6000, and 20% ethylene glycol) for a few seconds and flash‐frozen in liquid nitrogen for further data collection.

The diffraction data were collected using a single crystal at 100 K with a wavelength of 1.542 Å on an in‐house Oxford Diffraction Xcalibur Nova diffractometer. The data were processed by CryAlis Pro.^[^ ^33^ ^]^ The structure was solved by molecular replacement using the previously reported structure (PDB ID: 6LU7) as a model in Molrep.^[^ ^34^ ^]^ The statistics of the data collection and structural refinement are listed in Table S2, Supporting Information. The coordinates and refined structural factors of the complex have been deposited in the PDB under accession code 7VIC. Structural alignments and graphical representations were generated using PyMol (https://pymol.org).

##### Antiviral and Cytotoxicity Assay of Oridonin

Vero E6 cells were seeded in 96‐well plates and cultured overnight. To measure cytotoxicity, different concentrations (100 μL) of compound were added to the Vero E6 cells and incubated for 48 h at 37 °C, followed by cell viability assay with CCK‐8 reagent. The cell viability curves and CC_50_ were calculated using GraphPad Prism 7.0.

In the antiviral activity assay, Oridonin (10 mmol) or GC376 (10 mmol) in DMSO was diluted from 25 to 0.05 μmol with Dulbecco's Modified Eagle Medium (DMEM) containing 1% fetal bovine serum (FBS), and 1% DMSO‐containing medium was used as negative control. The Vero E6 cells were infected with SARS‐CoV‐2 virus at a MOI of 0.01. After approximately 2 h of incubation, the medium including virus was subsequently removed, fresh medium containing compounds at the above concentrations were added, and the cells were continuously cultured for 48 h. The viral RNA was extracted from the supernatants of cultured cells and qRT‐PCR was used to measure the SARS‐CoV‐2 RNA copy numbers. The following primers were used for quantitative PCR: ORF1ab‐F: 5′‐AGAAGATTGGTTAGATGATGATAGT‐3′, ORF1ab‐R: 5′‐TTCCATCTCTAATTGAGGTTGAACC‐3′, and Probe: 5′‐FAM‐TCCTCACTGCCGTCTTGTTGACCA‐BHQ1‐3′.

The SARS‐CoV‐2 RNA copy number in the presence of different concentrations of compounds was divided by that of the blank control to calculate the percentage inhibition or percentage relative RNA copy numbers. Inhibition curves were plotted by fitting the log (inhibitor) versus response (three parameters) mode with GraphPad Prism 7.0, and the EC_50_ was also generated in the table of results by GraphPad Prism 7.0. All of the infection experiments were performed at biosafety level‐3 (BSL‐3).

## Conflict of Interest

The authors declare no conflict of interest.

## Supporting information

Supplementary Material

## Data Availability

Coordinates and structure factors have been deposited in the Protein Data Bank for the SARS‐CoV‐2 3CLpro in complex with Oridonin under accession code PDB 7VIC.
