# Supplementary material for: Oridonin Inhibits SARS‐CoV‐2 by Targeting Its 3C‐Like Protease
Source: Small Sci. 2022 Mar 13;2(6):2100124. doi: 10.1002/smsc.202100124 (PMC9111243; doi:10.1002/smsc.202100124)
Supplement: Supplementary file 1 — Supplementary Material [file SMSC-2-2100124-s001.pdf]

Supporting Information

Oridonin inhibits SARS-CoV-2 by targeting its 3CL protease

Baisen Zhong[#], Weiyu Peng[#], Shan Du, Bingyi Chen, Yajuan Feng, Xinfeng Hu, Qi Lai, Shujie Liu, Zhong-Wei Zhou, Pengfei Fang, Yan Wu, Feng Gao [*], Huihao Zhou[*], and Litao Sun [*]

Supplementary Figures

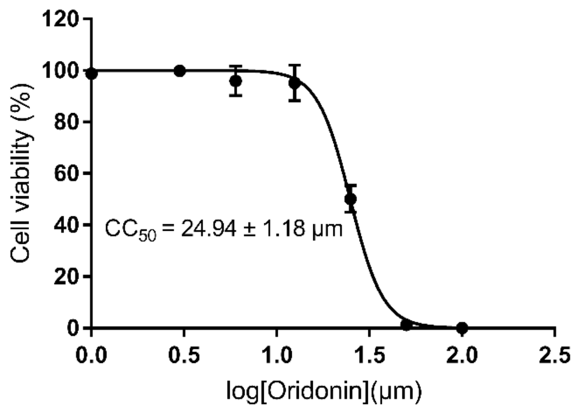

Supplementary Figure 1. The Cytotoxicity assay of Oridonin

The cytotoxicity of Oridonin on Vero E6 cells was measured by CCK-8.

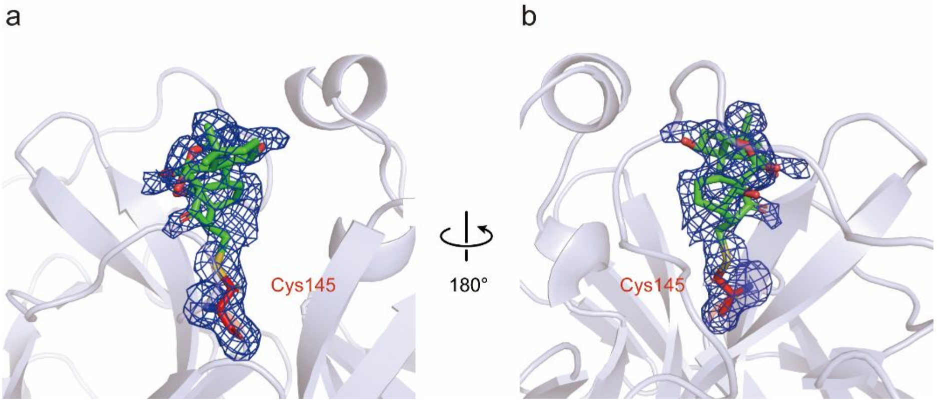

Supplementary Figure 2. Omit map of 3CLpro in complex with Oridonin

(a-b) In an enlarged view of the Ori-binding pocket, the Omit map is shown between Cys145 and Oridonin allowing to place an exact C-S covalent bond between the carbon atom on olefins of Oridonin and the sulfur atom of Cys145. The Fo-Fc contoured at 2.0σ is shown around Oridonin and Cys145(blue mesh).

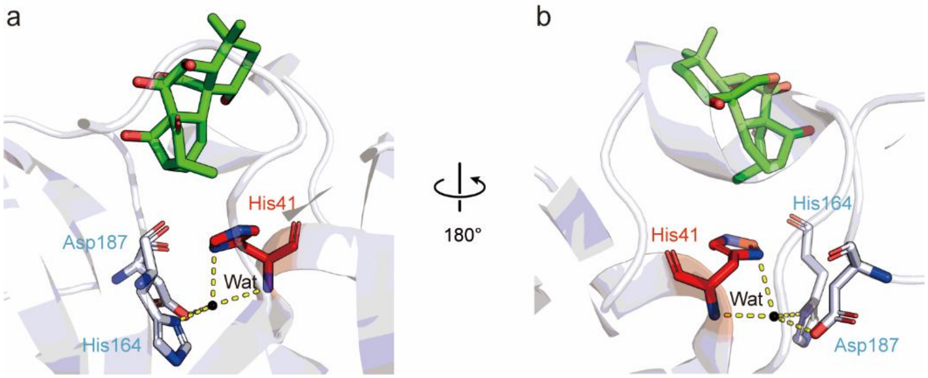

Supplementary Figure 3. Oridonin binds to 3CLpro without destroying the “catalytic water”

(a-b) The “catalytic water” (Wat) is shown in black spherical model, and the residues His41, His164 and Asp187 interacting with the “catalytic water” is shown in sticks. Oridonin is shown as green sticks.

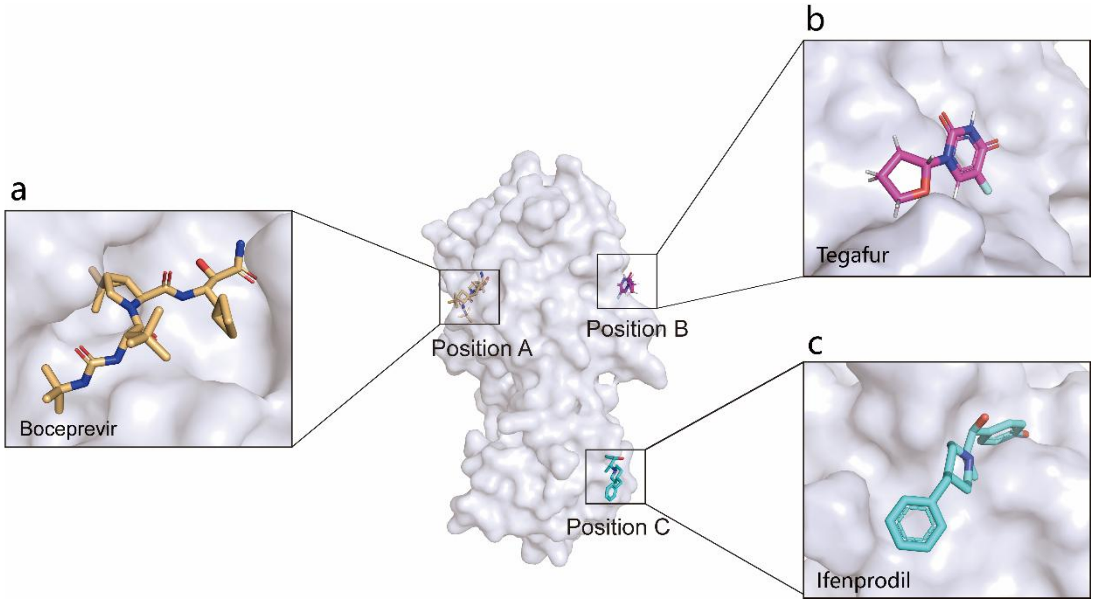

Supplementary Figure 4. Reported binding positions of 3CLpro

There are three ligand binding positions of 3CLpro. **(a)** position A, shown binding with Boceprevir. **(b)** position B in the opposite side of the position A, shown binding with Tegafur. **(c)** position C, shown binding with Ifenprodil.

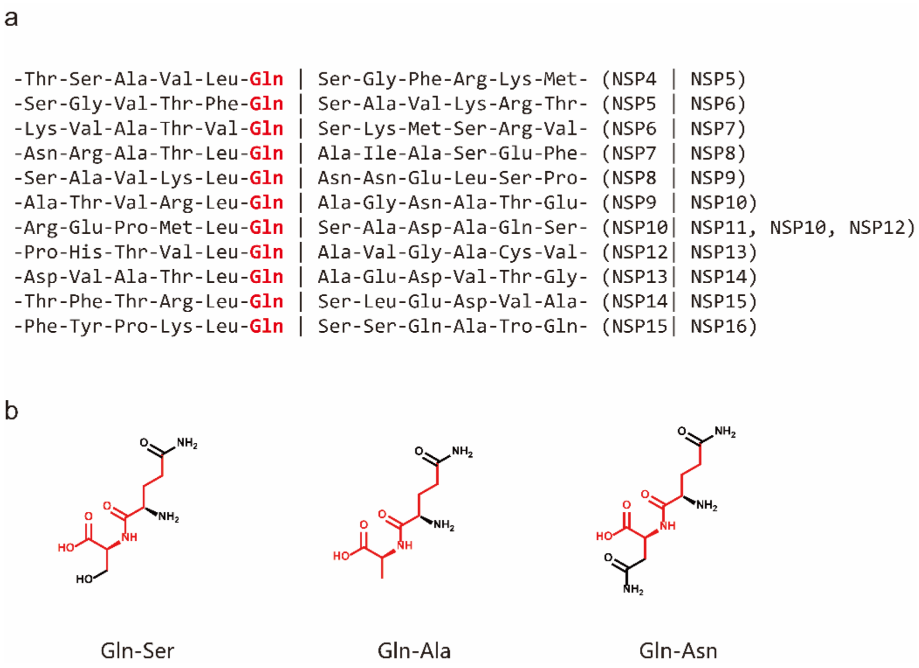

Supplementary Figure 5. 3CLpro proteolysis recognition site and structural scaffold of peptidomimetic inhibitions

(a) Substrate proteolysis site recognized by 3CLpro. Their N-terminals are all conservative Glutamine, and the C-terminals are Asparagine, Alanine or Serine. **(b)** The chemical structural scaffold of the amino acid dipeptide at the above conservative proteolysis site of 3CLpro substrates are shown in red.

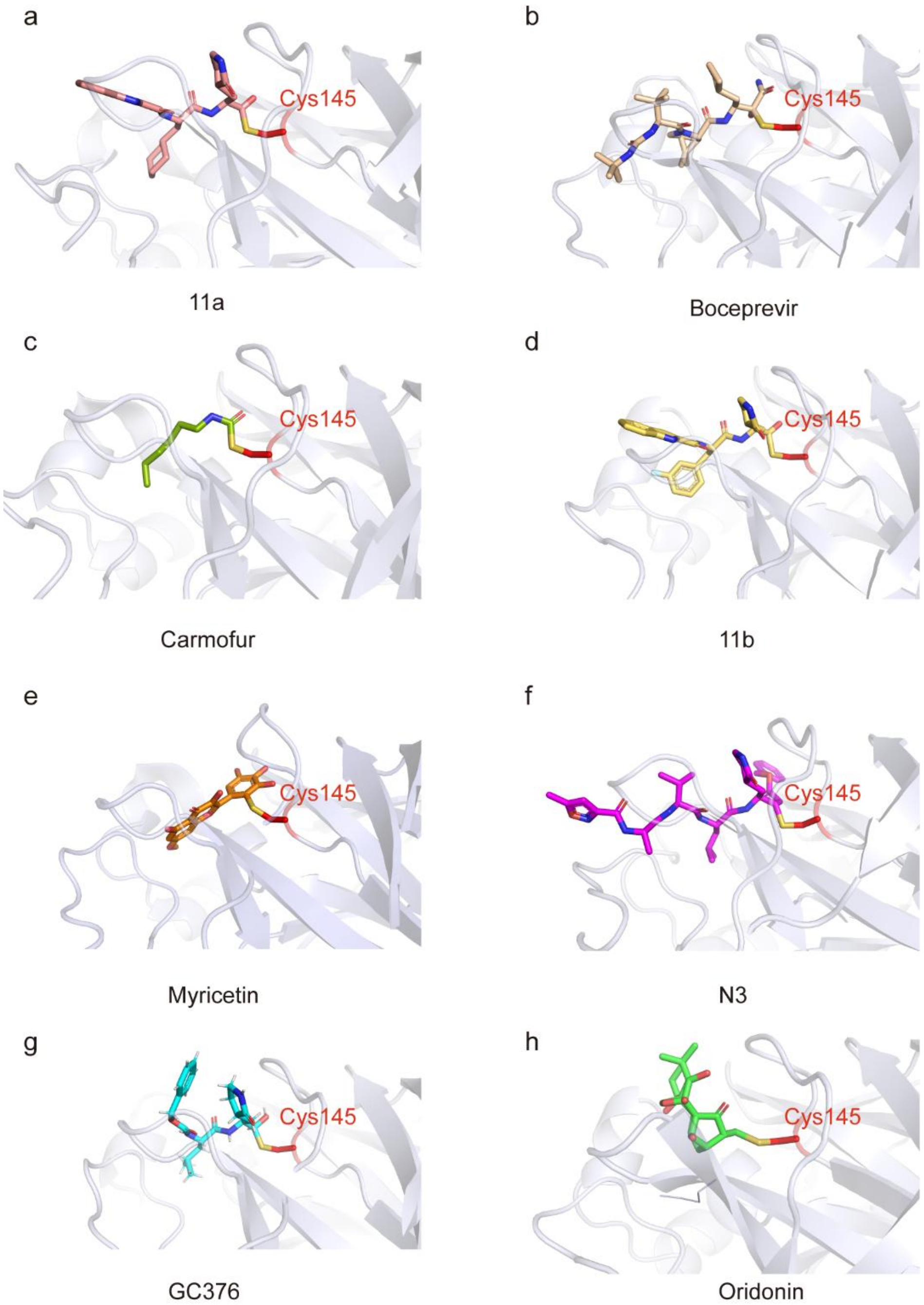

Supplementary Figure 6. Binding mode of Covalent binding inhibitors of 3CLpro

(a-h) Covalent binding inhibitors occupy the substrate-binding pocket by forming a covalent bond with Cys145 of 3CLpro.

Supplementary Table 1. The ligands of 3CLpro bound in different positions

Binding position	Ligand				
Position A	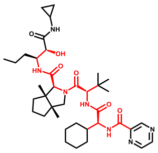<br>Telaprevir	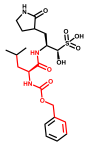<br>GC376	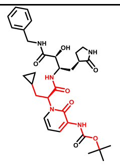<br>13b	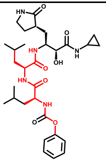<br>UAW248	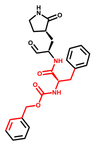<br>UAW247
	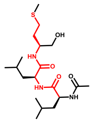<br>Boceprevir	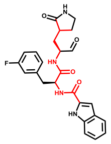<br>11b	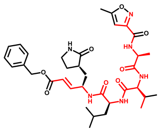<br>N3	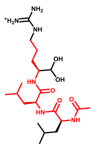<br>Leupeptin	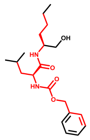<br>Calpeptin
Position B	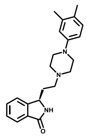<br>PD168568	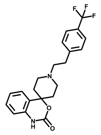<br>RS102895	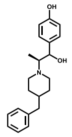<br>Ifenprodil	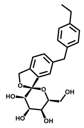<br>Tofogliflozin	
Position C	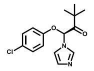<br>Climbazole	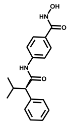<br>AR-42	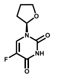<br>Tegafur	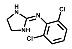<br>Clonidine	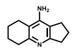<br>Ipidacrine

Supplementary Table 2. Data collection and refinement statistics

	3CLpro-Oridonin
**PDB Code**	7VIC
**Data collection**	
Resolution (Å)	24.49-2.10 (2.18-2.10)
Space group	I121
Cell dimensions	
a, b, c (Å)	52.18, 81.41 91.22
α, β, γ (°)	90.00, 96.14, 90.00
Wavelength (Å)	1.542
Unique reflections	22125 (2204)
R_{merge} (%)	0.129 (0.582)
Average I/σ(I)	10.5 (3.6)
Completeness (%)	99.7 (100.0)
Redundancy	4.4 (4.5)
$CC_{1/2}$	0.991 (0.814)
**Refinement**	
Resolution (Å)	24.50-2.10
Reflections for refinement/test	20990/1554
R_{work}/R_{free}	0.215/0.240
No. atoms	
Protein	2295
Ligand	26
Water	107
Mean B-factor ($Å^2$)	31.3
R.m.s. deviations	
Bond lengths (Å)	0.002
Bond angles (°)	1.2
Ramachandran plot (%)	
Favored	94.98
Allowed	4.69
Outliers	0.33

*) Values in parentheses are for the highest resolution shell.
